# Cost-effectiveness of integrated maternal HIV, syphilis, and hepatitis B screening opt-out strategies in Nepal: a modelling study

**DOI:** 10.1016/j.lansea.2024.100524

**Published:** 2025-01-10

**Authors:** Lucie Sabin, Kasim Allel, Ghanshyam Gautam, Naomi Saville, Hassan Haghparast-Bidgoli

**Affiliations:** a Institute for Global Health, University College London, London, United Kingdom; b Health Economics Research Centre (HERC), University of Oxford, London, United Kingdom; c HERD International, Kathmandu, Nepal

**Keywords:** Economic evaluation, Sexually transmitted disease, Integrated antenatal screening, Nepal, Modelling

## Abstract

**Background:**

The World Health Organisation (WHO) developed a comprehensive framework encouraging an integrated approach to achieve triple elimination of vertical transmission of HIV, syphilis, and hepatitis B in Asia. Current screening practices in Nepal show significantly lower coverage for syphilis and hepatitis B compared to HIV suggesting potential for integration. In this study, we aimed to model the cost-effectiveness of triple screening during antenatal care in Nepal.

**Methods:**

We modelled maternal HIV, hepatitis B, and syphilis cascade of care and their corresponding disease states using disease-specific Markov models over a 20-year horizon with a cycle length of one year. We compared dual integrated screening for HIV and syphilis and triple integrated screening for HIV, syphilis, and hepatitis B with HIV screening only. Costs were estimated from a provider's perspective. Results were presented as incremental cost-effectiveness ratios (ICERs). Univariable and probabilistic sensitivity analyses were conducted.

**Findings:**

Our modelling analysis showed that dual-integrated screening for HIV and syphilis was highly cost-effective when compared to current strategy of screening for HIV only (ICERs of US$18). Triple-integrated antenatal screening for HIV, syphilis, and hepatitis B was highly cost-effective compared with dual-integrated strategy with an ICER of US$114. Moreover, 100% and 98% of the probabilistic sensitivity analysis estimates for dual- and triple-integrated screening were proven cost-effective, compared to HIV-only screening.

**Interpretation:**

Our results support WHO recommendations for implementing integrated triple antenatal screening in Nepal and Asia more broadly, aiming to reduce maternal and neonatal morbidity through early detection and intervention.

**Funding:**

No funding was reported.

**Research in context**

**Evidence before this study**

The World Health Organisation (WHO) developed a comprehensive framework encouraging an integrated antenatal screening approach to achieve triple elimination of vertical transmission of HIV, syphilis, and hepatitis B in Asia. However, evidence of the cost-effectiveness of this strategy remains limited in low- and middle-income countries. We searched PudMed on 3 September 2024 using the terms ‘economic evaluation’, ‘integrated antenatal screening’, ‘HIV’, ‘syphilis’, ‘hepatitis B’, ‘Asia’, combined with relevant Boolean operators and with no date or language restrictions. We found only one study evaluating the cost-effectiveness of triple-integrated antenatal screening in Cambodia. Yet, no study was found elsewhere in the region.

**Added value of this study**

The current study provides the first evidence of the cost-effectiveness of dual-integrated screening for HIV and syphilis and triple-integrated screening for HIV, hepatitis B, and syphilis in Nepal compared with the current HIV-only approach. Focussing on the specific context of Nepal, it highlights the value of integrating services that can guide policymakers in designing interventions that consider cost, feasibility and health outcomes. This study contributes to advancing the debate on integrating public health strategies to maximise the effectiveness of maternal and child health programmes.

**Implications of all the available evidence**

Our results support WHO recommendations for the implementation of integrated triple antenatal screening in Asia, highlighting the need for further economic analyses of integrated screening pilots to inform decision-making. The implementation could reduce healthcare costs and improve integrative decision-making for population health.

## Introduction

Every year, a significant number of infants are born with HIV, syphilis, and hepatitis B, transmitted by their mothers during pregnancy or childbirth. In 2020, an estimated 150,000 children were newly infected with HIV worldwide.^1^ Meanwhile, the World Health Organisation (WHO) estimated that, in 2022, there were 700,000 cases of congenital syphilis, resulting in 150,000 early foetal deaths and stillbirths and 70,000 neonatal deaths globally.^2^ In addition, an estimated 240 million people were infected worldwide with hepatitis B in 2016 and most chronic infections are attributable to vertical transmission.^3^

The WHO developed a comprehensive framework for the triple elimination of vertical transmission of HIV, syphilis, and hepatitis B in Asia.^4^ This initiative aims to achieve the elimination of these three infections in newborns and infants by 2030 in Asia through integrated screening and timely treatment strategies to prevent vertical transmission. HIV screening coverage is often much higher than that for syphilis and hepatitis B, and integrated rapid screening technologies offer a unique opportunity to fill these gaps and improve the coverage and effectiveness of antenatal screening services. Given that all three infections can be detected by blood tests, integrating their screening into a single protocol may reduce demand and supply-side barriers to antenatal screening, including the logistical, time, and financial barriers associated with multiple screening for both women and health workers.^5^ The effectiveness of this approach has the potential not only to improve adherence to screening but also to optimise the use of resources within healthcare systems.

In Nepal, although the prevalence of these infections is relatively low compared with neighbouring countries, vertical transmission of these infections continues to be a critical public health concern.^6^ In 2020, HIV prevalence among adults aged 15–49 in Nepal was estimated at 0.12%.^7^ Hepatitis B prevalence was estimated at 5% among pregnant women in 2019.^8^ Syphilis prevalence estimates vary, with an estimated 1.5% among a group of 3570 women screened^9^ and 0.2% among 1362 women screened.^10^ Antenatal screening for HIV and syphilis is incorporated into national guidelines but faces barriers primarily from the supply side, such as a lack of trained healthcare providers, and logistical issues.^11^ Although antenatal screening for HIV has shown considerable improvements over the last decade, with 82% coverage among pregnant women reported by healthcare facilities in 2022,^12^ antenatal screening for syphilis and hepatitis B remains particularly low. In 2017, syphilis screening during pregnancy in Nepal was estimated at 0.3%.^13^ This makes it difficult to monitor infections on a national scale, and the burden of these diseases might be underestimated.

Given the constraints of limited resources and the need for prioritisation, health economic evidence becomes crucial in determining the most cost-effective strategies for integrated antenatal screening. Although several studies investigated the cost-effectiveness of simple screening for HIV, syphilis, and hepatitis B, only a few studies investigated dual integrated antenatal screening for HIV and syphilis. These studies on dual integrated screening, conducted in different settings, concluded that screening interventions were cost-effective.^14^, ^15^, ^16^ As far as we know, only one study conducted in Cambodia investigated the cost-effectiveness of a triple screening strategy.^17^ The study concluded that triple-integrated screening is highly cost-effective.

Yet, gaps remain in understanding the cost-effectiveness of integrated screening strategies and no study has been previously conducted to determine the optimal approaches tailored to the Nepalese context. In this study, we aimed to model the cost-effectiveness of triple screening during antenatal care in Nepal.

## Methods

### Models structure, parameterisation, and assumptions

Maternal HIV, hepatitis B, and syphilis cascade of care and their corresponding disease states were modelled using disease-specific Markov models adapted from previous studies.^16^, ^17^, ^18^ Although the models were constructed separately for each condition (see next subsections), they shared common parameters including disease prevalences, incidence, test coverage, maternal and infant mortality rates, and probability of infant infection. Key parameters of these disease models, including disease prevalences and screening coverage, were adjusted based on the most recent data available to reflect the Nepalese context. We employed a cycle length of a year and evaluated models over 20 years, projecting outcomes and costs starting from childbirth for the infant model and from the time of screening (average age at pregnancy^19^) for the women's model. We considered the following adverse pregnancy outcomes: stillbirth, neonatal death, congenital syphilis in live-born infants, perinatal HIV infection, and perinatal hepatitis B infection. Foetal losses due to syphilis were considered but foetal losses because of HIV and hepatitis B were not considered. This decision was taken because of the relatively lower incidence of foetal loss directly attributable to HIV and hepatitis B compared with syphilis. Co-infections/reinfections were not incorporated into the model and risks of vertical transmission of HIV, syphilis, and hepatitis B and associated infant health outcomes were independent of one another. This simplification made it possible to model the transmission and results of each infection independently, to better understand the impact of each disease.

We assumed that screening is carried out once during pregnancy at the first antenatal care (ANC) visit following an opt-out strategy. The sensitivity and specificity of the tests were considered in the models. Treatment for HIV, syphilis, and hepatitis B should be initiated as early as possible in positive pregnant women. We thus assumed that diagnosed as infected women were given treatment from the first ANC visit (week 12)^20^ which corresponds to time zero (t = 0) in our models. As the retention of antiretroviral therapy (ART) in women was 99% in 2021 in Nepal,^21^ we assumed no discontinuity once the treatment had begun and that visits as part of the pregnancy were the only means of access to treatment and screening for infected women and children. Treatment could not be started later in life. Pre-exposure prophylaxis (PreP) uptake is particularly low in Nepal and was not considered in our model.^21^ We excluded from our model pregnant women who were already diagnosed and on ART before their pregnancy.^22^ Infant infections and deaths were estimated based on exposure to diseases in utero with a probability dependent on the status of the mother during pregnancy. Based on Nepal's protocol,^6^ we assumed that all children from positive mothers with known status were screened, regardless of the treatment status of the mothers, and adherence to children's treatment was independent of the mother's adherence.

Detailed models for each of the included diseases are presented below and schematics are presented in Supplementary Material (Appendix S1).

#### Markov model for HIV

Four disease states were considered for HIV: uninfected, HIV, AIDS, and death.^16^ The screening sequence considered in the model was the one recommended in the national guidelines namely a rapid test confirmed by a second one if positive.^6^ Regarding the ART treatment for HIV-positive pregnant women, it consisted of a once-daily fixed-dose combination of Tenofovir Disoproxil Fumarate (TDF), Lamivudine (3TC), and Efavirenz (EFV). ART should be continued lifelong^6^ so we assumed that pregnant women treated took the treatment for 20 years. According to the national guidelines,^6^ the treatment of babies at risk of transmission was dual prophylaxis and Nevirapine (NVP) for 12 weeks.

#### Markov model for syphilis

Four disease states were considered for syphilis: uninfected, early (primary/secondary) or latent syphilis, tertiary syphilis, and death.^16^ In the case of syphilis transmission, three adverse outcomes were considered: stillbirth, congenital syphilis, and neonatal death. The screening sequence considered in the model was the one recommended in the National Guidelines on Management of Sexually Transmitted Infections in Nepal^6^ knowing a rapid test confirmed by a treponema pallidum particle agglutination assay (TPPA) if positive. If tested positive, the recommendations differentiated treatment based on the stage of the syphilis: late (more than two years) or early (less than two years). This distinction was not made in our model, and we considered that when tested positive, women would be offered benzathine penicillin intramuscular in a single dose.^15^ In the case of congenital syphilis, the children would be treated with the same process.

#### Markov model for hepatitis B

Six disease states were considered for hepatitis B^18^: uninfected, chronic hepatitis B, active hepatitis B, cirrhosis including compensated and decompensated, hepatocellular, and death. The costs of vaccination for hepatitis B were not considered in our analysis as the focus was on integrated screening and treatment approaches rather than vaccination strategies. Moreover, hepatitis B vaccination is implemented as a routine practice in Nepal and its costs are already accounted for in public health budgets. In 2016, the Immunization Act 2072 BS recognised hepatitis B vaccination as the right of all Nepalese children below 15 months. We, therefore, assumed that none of the women of childbearing age had been immunised by vaccination. The screening sequence recommended in the national guidelines is a rapid test confirmed by an enzyme-linked immunosorbent assay (ELISA) test for hepatitis B surface antigen (HBsAg).^8^ If tested positive, the recommended treatment for the mother is Tenofovir Disoproxil Fumarate (TDF).^8^ We assumed a lifetime treatment for treated women. In the case of children with hepatitis B, they will be treated with Hepatitis B Immunoglobulin (HBIG).

### Data collection

Antenatal care-related data including the number of pregnant women per year, screening and treatment coverage as well as epidemiological data including the prevalence of HIV, syphilis, and hepatitis B among pregnant women were taken from the 2022 Nepal Demographic and Health Survey (NDHS), 2021 Nepal Health Facility Survey and governmental reports. Costs data, including screening and drug costs, were obtained from the Global Fund's pooled procurement mechanism reference prices, on which the Nepalese government bases its negotiations for the purchase of supplies. Health worker time costs were calculated based on the public sector scale.^23^ See Supplementary Material (Appendix S2, Table S1) for parameter details.

### Screening interventions

We compared three interventions with different screening interventions (Table 1): HIV screening only (status quo), HIV and syphilis integration (intervention 2), and HIV, syphilis, and hepatitis B integration (intervention 3).

**Table 1:** HIV, syphilis, and hepatitis B testing model interventions.

Intervention	Description
**Status quo** HIV screening only	Integration of ANC and screening only for HIV with HIV screening being offered at the first ANC visit.
**Intervention 2** Integrated screening of HIV and syphilis	Integration of HIV and syphilis activities into ANC routine visits, with the activities taking place in the same health facility.
**Intervention 3** Integrated screening of HIV, syphilis, and hepatitis B	Integration of HIV, syphilis, and hepatitis B activities into ANC routine visits, with the activities taking place in the same health facility.

Notes: ANC = Antenatal care. HIV = Human immunodeficiency virus. Integration = Conducting screening for several diseases into a single blood draw.

### Cost-effectiveness analyses

#### Outcomes

To assess the economic benefits of HIV, hepatitis B, and syphilis integrated screening and treatment, we analysed the investment costs and the impact on the population's health for each intervention. Key indicators for effectiveness included the number of new infections, the number of disease-related adverse events, the number of person-years living with the diseases and the number of diseases-related deaths. Investment costs included labour costs and the purchase of screening kits and treatments.

For each infection, disability-adjusted life years (DALYs) were calculated as the sum of years of life lost and years lived with a disability. Disability weights for the different health statuses were obtained from the Global Burden of Disease study.^24^ DALY for each stillbirth and neonatal death were equal to the years of life lost due to the death of the newborn, taking into account the local potential life expectancy if the child had survived. We assumed a 3% discount rate for DALYs.

#### Costs

Costs consisted of direct medical costs for screening and treatment, including the cost of screening kits, treatment and labour costs for screening, data entry, counselling, training, and the supply of drugs. See Supplementary Material (Appendix S2, Table S1) for parameter details. We excluded from the analysis the costs related to the infrastructure of health facilities, as well as distribution costs. We assumed that treatments were given during routine ANC visits and that no further visits were needed. Costs of stillbirths and neonatal deaths related to syphilis were considered the same and equal to the costs of delivery with complications. The costs of vertical transmission of HIV and hepatitis B taken into account are the costs of treating infected infants and mothers. When collected in 2024's local currency (Nepalese rupees), we converted costs to (United States dollars) US$ using the 2024 exchange rate.^25^ We applied a 3% annual discount rate for costs.^26^

#### Analysis

A cost-effectiveness analysis was conducted from a healthcare provider's perspective. Interventions 2 and 3 were compared with the status quo and the impact of integrative approaches on differences in life quality indicators were assessed. We calculated the incremental cost-effectiveness ratio (ICER) as the ratio of the incremental investment cost associated with implementing interventions 2 and 3 compared with the status quo, divided by the expected benefits to the population resulting from the improved screening coverage in these interventions. The incremental investment cost included the additional expenditure incurred in moving from the status quo to integrated approaches, while the expected population benefits included the DALYs. The determination of cost-effectiveness depended on whether the ICERs were below the country-specific cost-effectiveness threshold otherwise understood as the country's willingness to pay. Cost-effectiveness was determined by comparing ICERs to the country-specific cost-effectiveness threshold, which reflects the country's willingness to pay. Different thresholds were considered, ranging from 50% of gross domestic product (GDP) per capita (662 US$)^27^ as estimated by Wood et al.^28^ to three times GDP per capita (3972 US$) based on WHO guidelines.^29^

Analyses were conducted in Microsoft Excel 2023 and results were reported according to the Consolidated Health Economic Evaluation Reporting Standards (CHEERS) statement.^26^ Our models and analyses are available on the open-access repository UCL Discovery.

#### Sensitivity analysis

We conducted one-way sensitivity analyses to assess the effect of changing models’ parameters, including disease prevalence, test and treatment costs, discount rates. These parameters were chosen based on their potentially significant influence on the results, as identified in the existing literature, or their inherent uncertainty, which could lead to highly volatile results. We used low and high parameter values from confidence interval bounds when available, ±10% relative changes for cost parameters and ±20% relative changes for all other parameters.

In addition, we conducted a probabilistic sensitivity analysis (PSA) to allow several parameters to be varied simultaneously according to their probability distributions. Upper and lower bounds of parameters are presented in Appendix S2, Table S1. Probability distributions were assigned to each parameter based on available data and expert opinion. We assumed that probabilities follow a beta distribution and continuous variables a gamma distribution.^30^ We used Monte Carlo simulation techniques to generate 1000 iterations of the model and quantify the impact on the outcomes of interest.

## Results

We modelled populations of 752,506 pregnant women in Nepal.^31^ In our models, routinely offering HIV, syphilis, and hepatitis B rapid screening at the first ANC visit (intervention 3) was more effective compared with rapid dual screening for HIV and syphilis (intervention 2) or HIV screening only (status quo), it was also costlier (Table 2). Dual rapid diagnostic tests at the first antenatal care visit averted 470 congenital syphilis cases more than the status quo and reduced DALYs by 28,516 (16.12% reduction) compared to the status quo. Triple integrated screening strategy averted 479 vertical transmissions of hepatitis B more than in intervention 2 and reduced DALYs by 8166 (5.50% reduction compared to intervention 2). Per DALY averted, dual integrated screening for HIV and syphilis at the first antenatal care visit cost 18 US$ more than HIV screening, and triple integrated screening cost 40 US$ more than HIV screening only and 114 US$ more than dual integrated screening for HIV and syphilis. Dual-integrated screening was highly cost-effective compared to HIV screening only and triple-integrated screening was highly cost-effective compared to dual-integrated screening.

**Table 2:** Health effect, cost, and cost-effectiveness results of maternal HIV, syphilis, and hepatitis B screening interventions.

	Total cost (US$)	Total vertical transmission			Total DALYs	Incremental costs (US$)	Incremental DALYs averted	ICER (US$/DALY averted)
		HIV	Syphilis	Hepatitis B				
**Status quo** *HIV only*	1,782,350.19	83	783	941	176,920	Reference	Reference	Reference
**Intervention 2** *HIV and syphilis*	2,307,840.25	83	313	941	148,403	525,490	28,516	18
**Intervention 3** *HIV, syphilis, and hepatitis B*	3,237,353.89	83	313	462	140,237	929,514	8166	114

Notes: ICER = Incremental cost-effectiveness ratio. HIV = Human immunodeficiency virus. DALYs = Disability-adjusted life years. US$ = United States Dollars. Incremental costs, incremental DALYs averted, and ICER for intervention 2 were obtained by comparing dual-integrated screening with HIV screening only. Incremental costs, incremental DALYs averted, and ICER for intervention 3 were obtained by comparing triple-integrated screening with dual-integrated screening.

**Fig. 1:**
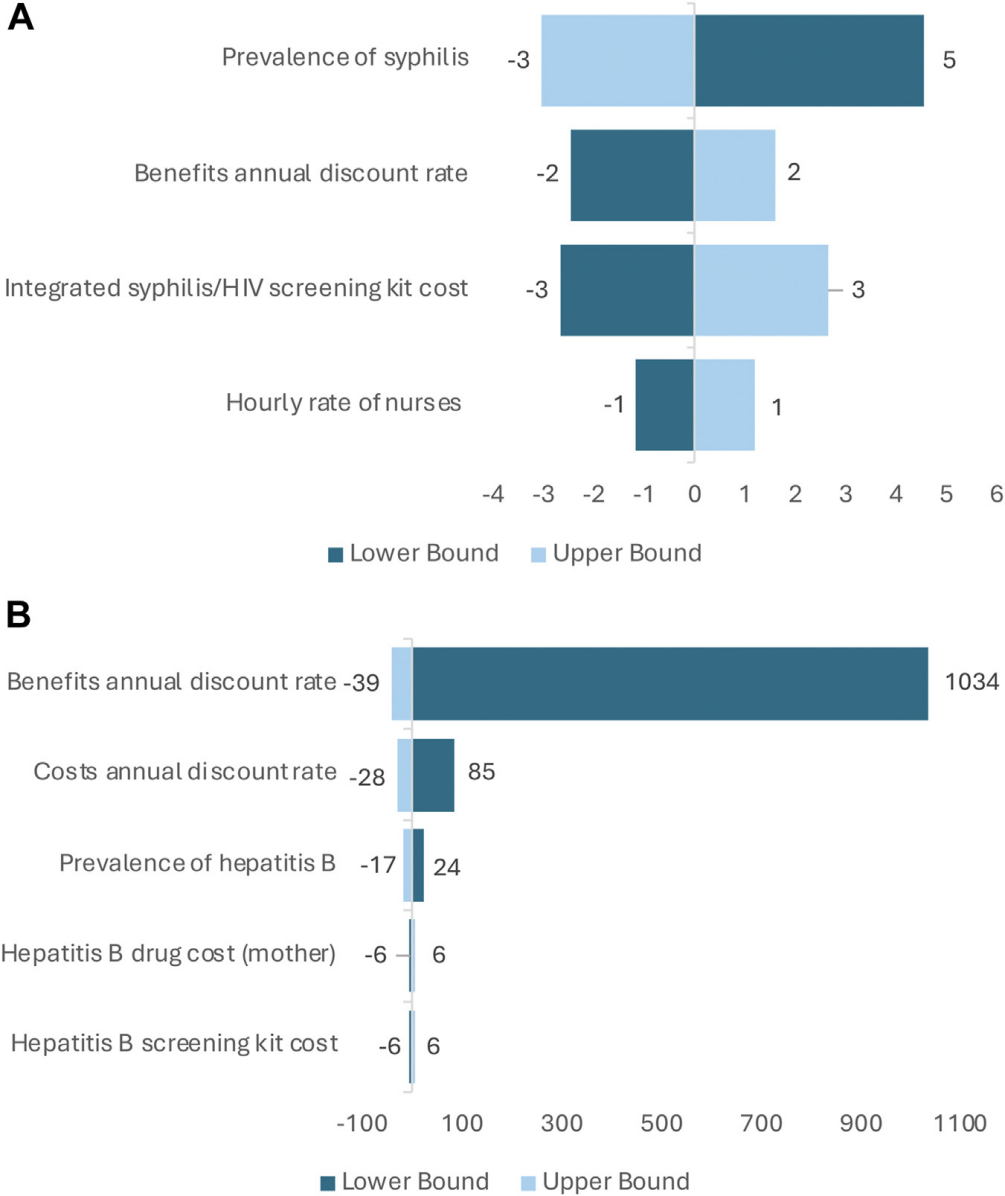
Tornado diagrams of the percentage change in the incremental cost-effectiveness ratio (ICER) for dual-integrated screening for HIV compared with HIV screening only (status quo) and syphilis and triple integrated screening for HIV, syphilis, and hepatitis B compared with dual-integrated screening produced from a deterministic one-way analysis of key parameters. **a.** Dual integrated screening for HIV and syphilis compared to HIV screening only. **b.** Triple-integrated screening for HIV, syphilis, and hepatitis compared to dual-integrated screening. Notes: Dark blue bars indicate the absolute change in the ICER when the given parameter is at its minimum plausible value, whereas light blue bars indicate the absolute change in the ICER when the same parameter is at its maximum plausible value. Parameters listed towards the top of the diagram contribute more to the overall uncertainty in the cost-effectiveness ratio than do those towards the bottom, which contribute relatively little to the uncertainty in the cost-effectiveness ratio. Parameters that do not contribute at all were not presented in these diagrams.

Fig. 1 identified the main factors influencing the ICER of dual screening for HIV and syphilis (intervention 2) and triple integrated screening (intervention 3). Detailed results of the one-way sensitivity analysis are presented in Appendix S3, Table S3. HIV and syphilis drug costs had a minimal impact on ICER (<1%, compared to status quo results). The ICER for dual integrated screening (intervention 2) was sensitive to the price of the integrated syphilis/HIV test kit, syphilis prevalence and hourly rate of nurses, maximum 14%, 25%, and 6% variations, respectively, compared to the status quo scenario. The ICER for triple integrated screening (intervention 3) fluctuated between −5% and 5%, compared to the status quo scenario, due to the cost of the hepatitis B test kit and maternal drugs. Both scenarios were highly sensitive to the annual discount rate of benefits. In addition, the ICER for triple integrated screening (intervention 3) was highly sensitive to the prevalence of hepatitis B (−15% and 21% variations from the status quo scenario).

**Fig. 2:**
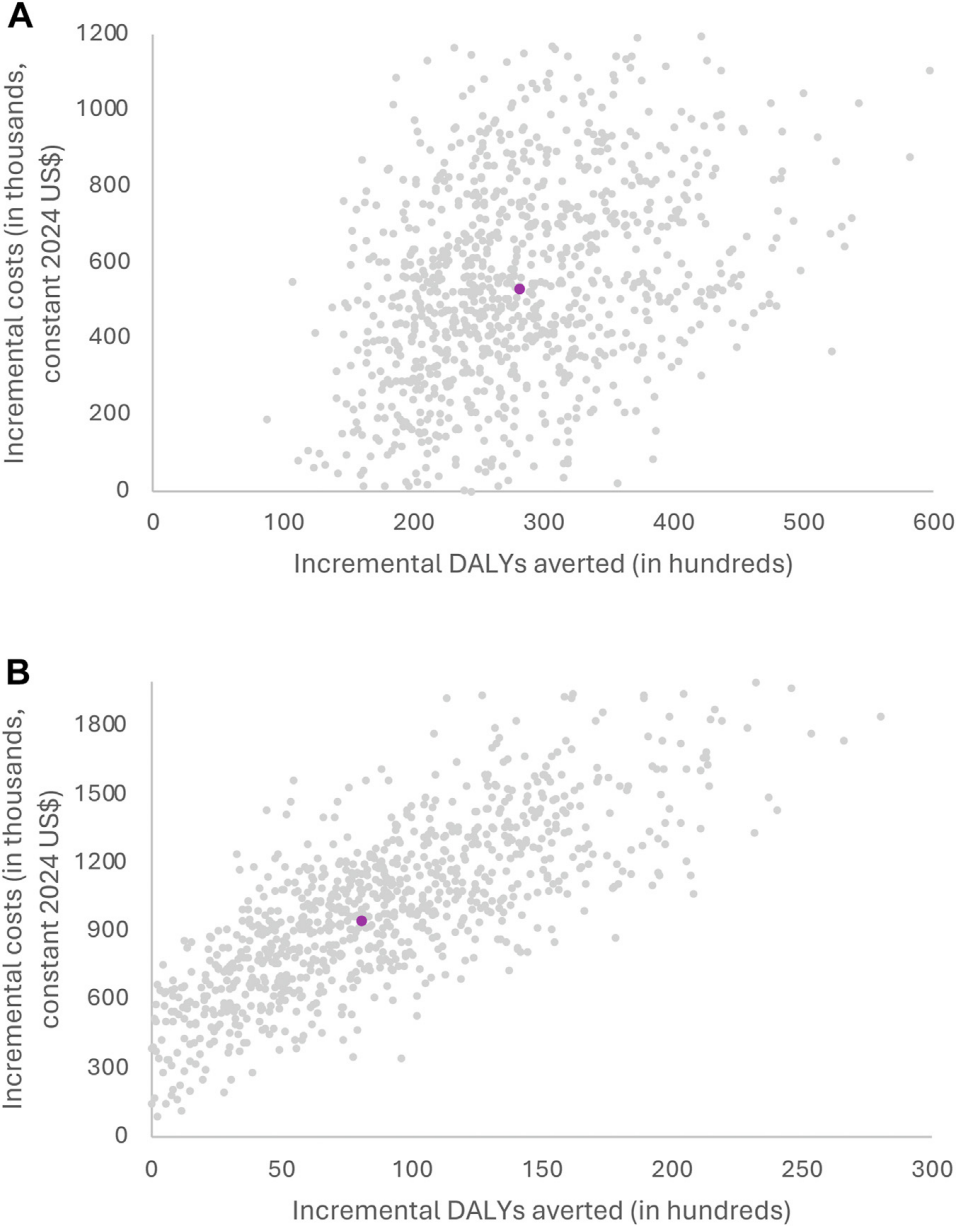
Cost-effectiveness plane showing the statistical uncertainty around estimates of incremental costs and incremental DALYs averted for dual-integrated screening for HIV compared with HIV screening only (status quo) and syphilis and triple-integrated screening for HIV, syphilis, and hepatitis B compared with dual-integrated screening. **a.** Dual integrated screening for HIV and syphilis compared to HIV screening only. **b.** Triple-integrated screening for HIV, syphilis, and hepatitis compared to dual-integrated screening. Notes: Each grey dot represents the results of one of the 1000 simulations. The incremental cost-effectiveness ratio (ICER) for each simulation is defined as the slope of the line from the origin to that data point. The pink dot represents the mean incremental costs for the 1000 simulations of 532,608 US$ [95% CI: 514,418; 550,734] and 946,744 US$ [95% CI: 919,960; 973,528] and a mean of 28,249 [95% CI: 27,725; 28,773] and 8053 [95% CI: 7665; 8441] DALYs averted for Intervention 2 and 3 respectively, giving ICER mean ratios of 19 US$ [95% CI: 18; 20] and 118 US$ [95% CI: −91; 326]. Of the 1000 estimates for integrated double screening, 96.3% were in the right upper quadrant and 3.7% in the right lower quadrant. For triple integrated screening, out of 1000 simulations, 91.1% were in the upper right quadrant, 1.2% were in the lower left quadrant, 7.6% were in the upper left quadrant, and 1.0% were in the lower right quadrant.

**Fig. 3:**
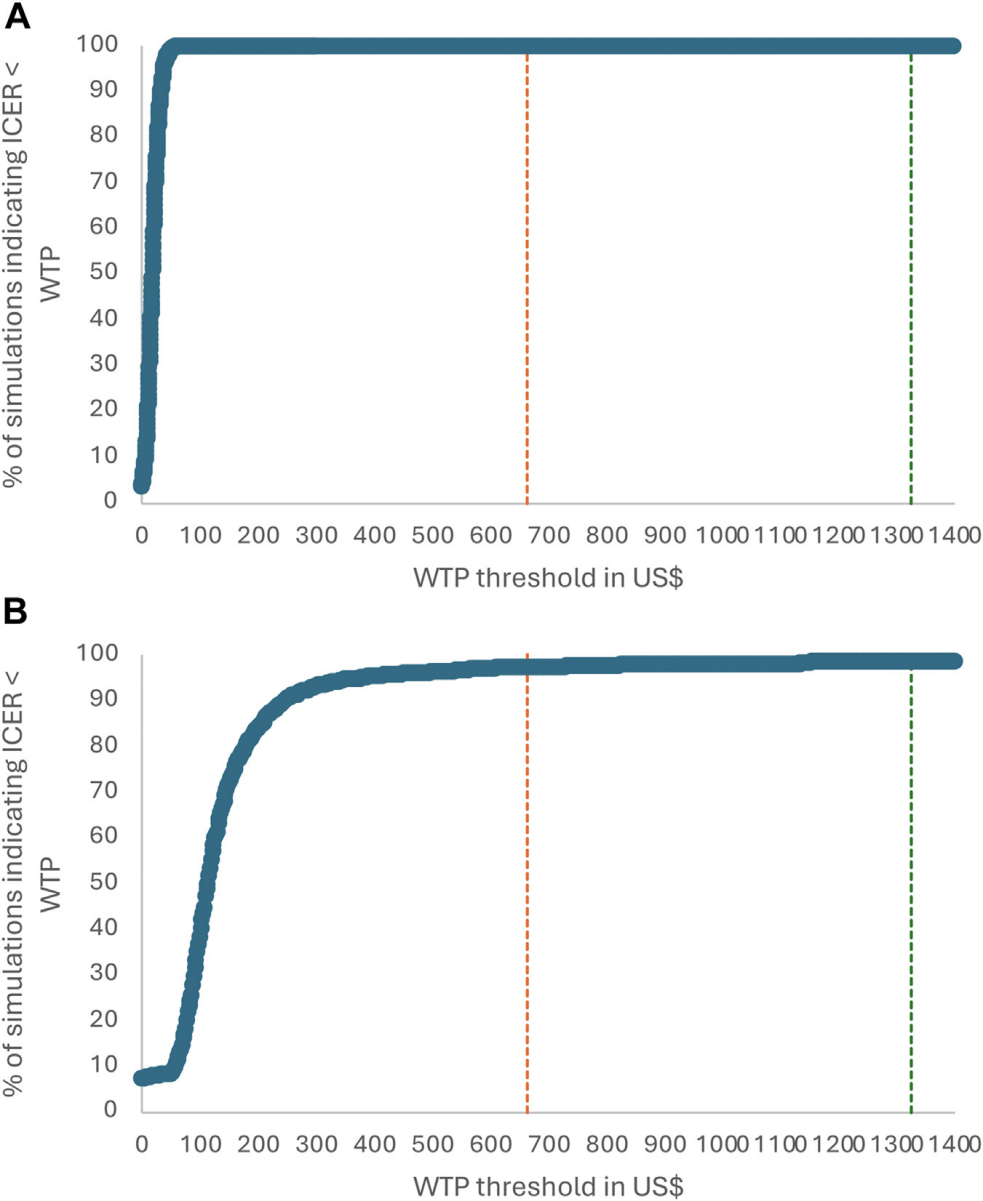
Cost-effectiveness acceptability curves, by intervention. **a.** Dual integrated screening for HIV and syphilis compared to HIV screening only. **b.** Triple-integrated screening for HIV, syphilis, and hepatitis compared to dual-integrated screening. Notes: Dashed vertical lines represent different acceptability thresholds. The orange one is equal to 50% of the Nepalese gross domestic product (GDP) per capita (662 US$) and the green one is equal to one time the Nepalese GDP per capita (1324 US$).

In PSA (Fig. 2), the mean incremental cost per DALY averted was respectively 19 US$ and 118 US$ for dual and triple integrated screening (95% CI: 18–20 for intervention 2 and −91 to 326 for intervention 3). Fig. 3 presents the cost-effectiveness acceptability curves of the interventions. At a willingness-to-pay of 662 US$,^28^ 100% and 98% of the PSA estimates for dual and triple-integrated screening, respectively, remained cost-effective. With a willingness to pay equal to three times Nepal's GDP per capita (3972 US$),^29^ 100% of the PSA estimates for both dual- and triple-integrated screening remained cost-effective.

## Discussion

Although Nepal has a concentrated epidemic, it is still far from achieving the triple elimination targets set by the WHO.^32^ In 2020, only 65% of pregnant women had a known HIV status, and coverage of syphilis and hepatitis B screening among women receiving antenatal care was 0% in 2018, well below the 95% target.^32^ This highlights the importance of promoting an integrated opt-out strategy for HIV, syphilis, and hepatitis B screening during pregnancy to achieve universal screening as part of routine antenatal care practice. Our modelling analysis demonstrated the high cost-effectiveness of integrated antenatal dual screening for HIV and syphilis compared with the HIV-only screening strategy currently implemented in Nepal. It also demonstrated the high cost-effectiveness of triple-integrated screening for pregnant women and children compared with dual-integrated strategy.

Our study indicated that triple screening was cost-effective compared with dual testing for HIV and syphilis and its implementation seems relevant in the Nepalese context. The ICER was found to be highly sensitive to disease prevalence, with a 20% increase in syphilis prevalence leading to a 16% decrease in the ICER for dual-integrated screening and a 20% increase in hepatitis B prevalence leading to a 15% decrease in the ICER for triple-integrated screening. While these variations highlight the importance of accounting for global spatial disparities in the prevalence of diseases such as HIV, syphilis, and hepatitis B, the PSA analyses—including these parameters—indicated that at a willingness-to-pay threshold of 662 US$, integrated dual and triple screening remained cost-effective in 100% and 98% of simulations, respectively.

The ICER for triple-integrated screening was found to be highly sensitive to the coverage of hepatitis B screening and treatment, suggesting a potential reduction in costs with increased coverage. This suggests that increased coverage could enable fixed costs to be spread more effectively, thereby reducing the cost per DALY averted. These results, together with the important role of health workers in antenatal screening in Nepal^11^ highlight the importance of improving screening coverage. More specifically, a 10% reduction in the cost of hepatitis B screening kits led to a 5% reduction in ICERs for triple-integrated screening. Integrated rapid screening kits would allow costs to be shared between the three diseases. Assuming that costs are similar to those of current dual screening kits for HIV and syphilis, costs per disease for hepatitis B could potentially be reduced by 47%, leading to a potential reduction in triple-integrated screening ICER of over 27%.

Integration offers significant potential for time savings for health workers. This time saving could enable health workers to devote their time to other interventions. However, it is essential to ensure that the quality of services is maintained through appropriate supervision and training of health workers. In our study, the benefits of integration may be underestimated since we did not consider the time saved by pregnant women. This time saved could increase the acceptability of the test and improve their access to antenatal care services, given that long waiting times are frequently reported in Nepal.^11^

A previous qualitative study in Nepal found that the availability of blood tests for STI screening is limited in some health facilities.^11^ In addition, only 22% are equipped for syphilis screening.^33^ Promoting the use of integrated rapid tests in Nepal could alleviate this problem. Rapid tests require minimal training and laboratory infrastructure.^34^ Based on integrated rapid-test approaches, Thailand's and Sri Lanka's models have been recognised by WHO as having reached the elimination targets for the vertical transmission of HIV and syphilis.^35^^,^^36^

We compared our findings with other economic evaluations evaluating double and triple-integrated antenatal screening. By supporting antenatal dual- and triple-integrated screening, our results are in line with other studies that have attempted to assess the cost-effectiveness of universal screening strategies for pregnant women.^15^, ^16^, ^17^ A cost-effectiveness analysis of maternal dual rapid diagnostic tests at the first antenatal care visit compared with individual HIV and syphilis tests, using modelling methods for four countries (Kenya, Colombia, South Africa, and Ukraine), found that incorporating dual rapid diagnostic tests into antenatal care can be cost-effective in countries with varying HIV prevalence.^15^ Similarly, a modelling-based cost-effectiveness analysis in China compared four screening strategies—no screening, HIV screening only, syphilis screening only, and HIV and syphilis screening—and concluded that antenatal HIV screening programmes that include syphilis screening are likely to be significantly more cost-effective than HIV screening alone and to prevent significantly more adverse pregnancy outcomes.^16^ In Cambodia, a cost-effectiveness evaluation of integrated triple testing concluded that integration of screening for HIV, syphilis, and hepatitis into antenatal, perinatal and postnatal care is highly cost-effective and efficient.^17^

Although disease-specific funding allows considerable progress to be made, as was the case with HIV for example,^37^ it often limits the ability of programmes to tackle several diseases simultaneously, which is a source of inefficiency. This narrow focus can lead to inefficient use of resources, redundant efforts and the loss of economies of scale that integrated approaches can achieve. In the case of antenatal screening, focussing on screening for specific diseases can lead to missed opportunities to address other co-existing conditions, particularly in settings where resources are limited and several diseases may have overlapping risk factors and target populations. Integrated screening also enables a patient-centred approach that many health systems and ministries of health are striving to implement.^38^ Integrated approaches may also offer a more sustainable and effective public health model, and the extension of screening efforts within maternal and child health programmes could potentially include other conditions as testing, laboratory, and treatment capacity improves. Our results encourage a gradual extension of Nepal's newborn screening programme to multiple other conditions as health infrastructure and economic viability improve.

This study has some limitations which must be considered when interpreting the results. Firstly, we did not take account of foetal losses due to HIV and hepatitis B. In addition, we did not include co-infections or reinfections in our models, and the risks of vertical transmission of HIV, syphilis, and hepatitis B, as well as associated infant health outcomes, were treated independently. This could potentially lead to an underestimation of the true health impact. Given the low number of reported cases in Nepal, we did not take account of follow-up losses. Variations in disease prevalence and testing coverage in different subsets of the population were not considered. Screening and treatment protocols were assumed to be based on national guidelines, without considering variations in clinical practice between healthcare facilities. Furthermore, our analysis is sensitive to disease prevalence. Given limited availability of regional prevalence data in Nepal, our study aimed to provide a generalisable assessment of integrated antenatal screening on a national scale. However, our results should be interpreted with caution in contexts where prevalence rates differ considerably. The study assumed immediate initiation of treatment at the first ANC visit, which may not reflect actual delays in diagnosis and initiation of treatment. Screening and treatment of children were assumed to be independent of their mothers' adherence to treatment, which may not accurately reflect real-world scenarios. Limited access to cost data at the national level forced us to make assumptions based on international costs. Finally, the simplified cost calculations excluded certain costs, such as infrastructure and supply costs, which could lead to an underestimation of total costs. Despite these limitations, our results suggest the potential cost-effectiveness of triple antenatal integrated screening and the potential for reaching triple elimination of vertical transmission. A pilot economic evaluation would enable us to test effectiveness, determine costs, and eliminate assumptions from our model. It is essential to design the pilot project carefully to adapt to the context to exploit its full potential.^39^

### Conclusion

In conclusion, we found the high cost-effectiveness of the implementation of integrated HIV, hepatitis B, and syphilis screening into routine ANC services for pregnant women and children in Nepal compared with the current dual-integrated approach for HIV and syphilis. The current development of integrated rapid screening kits for HIV, syphilis, and hepatitis B will reinforce the feasibility and practicality of adopting this integrated approach. Successful integration requires effective and high-quality ANC services, supported by necessary human resources, facilities, and supplies. Given heavy long-life treatments necessary for hepatitis B and HIV-positive individuals, early diagnosis and prevention are essential to ensure timely treatment and eliminate vertical transmission. Our results support WHO recommendations for the implementation of integrated triple antenatal screening in Asia, highlighting the need for further economic analyses of integrated screening pilots to inform decision-making.

## Contributors

LS-conceptualisation, methodology, investigation, formal analysis, visualisation, writing—original draft; KA-methodology, writing—review and editing; GG-methodology, writing—review and editing; NS-supervision, writing—review and editing; HHB-supervision, methodology, writing—review and editing; LS, KA, GG, and HHB have directly accessed and verified the underlying data reported in the manuscript. The authors were not precluded from accessing data in the study, and they accept responsibility for submitting it for publication.

## Data sharing statement

All data relevant to the study are included in the article or uploaded as Supplementary Information. Our models and analyses are available on the open-access repository UCL Discovery.

## Declaration of interests

Authors have no conflict of interest to declare.

## References

[1] WHO (2021).

[2] WHO (2024). Syphilis fact sheets. https://www.who.int/news-room/fact-sheets/detail/syphilis#:%7E:text=population%20(1).-,Transmission,to%202%20years%20after%20infection.

[3] Mohsen W., Levy M.T. (2017). Hepatitis A to E: what's new?. Intern Med J.

[4] WHO (2018).

[5] Sabin L., Haghparast-Bidgoli H., Miller F., Saville N. (2024). A systematic review of barriers and facilitators to antenatal screening for HIV, syphilis or hepatitis B in Asia: perspectives of pregnant women, their relatives and health care providers. PLoS One.

[6] National Centre for AIDS and STD Control (2022).

[7] UNAIDS (2020).

[8] Ministry of Health and Population (2023).

[9] Upreti P. (2019). Prevalence of STIs among nepalese women population. Sex Transm Infect.

[10] Shakya S., Thingulstad S., Syversen U. (2018). Prevalence of sexually transmitted infections among married women in rural Nepal. Infect Dis Obstet Gynecol.

[11] Sabin L., Haghparast-Bidgoli H., Bhattarai S. (2024). A qualitative study investigating factors influencing the implementation of integrated screening for HIV, syphilis, and hepatitis B for pregnant women in Nepal. PLOS Glob Public Health.

[12] NCASC (2022).

[13] Trivedi S., Taylor M., Kamb M.L., Chou D. (2020). Evaluating coverage of maternal syphilis screening and treatment within antenatal care to guide service improvements for prevention of congenital syphilis in countdown 2030 countries. J Glob Health.

[14] Newman Owiredu M., Newman L., Nzomo T. (2015). Elimination of mother-to-child transmission of HIV and syphilis: a dual approach in the African Region to improve quality of antenatal care and integrated disease control. Int J Gynecol Obstet.

[15] Rodriguez P.J., Roberts D.A., Meisner J. (2021). Cost-effectiveness of dual maternal HIV and syphilis testing strategies in high and low HIV prevalence countries: a modelling study. Lancet Glob Health.

[16] Owusu-Edusei K., Tao G., Gift T.L. (2014). Cost-effectiveness of integrated routine offering of prenatal HIV and syphilis screening in China. Sex Transm Dis.

[17] Zhang L., Tao Y., Woodring J. (2019). Integrated approach for triple elimination of mother-to-child transmission of HIV, hepatitis B and syphilis is highly effective and cost-effective: an economic evaluation. Int J Epidemiol.

[18] Su S., Wong W.C., Zou Z. (2022). Cost-effectiveness of universal screening for chronic hepatitis B virus infection in China: an economic evaluation. Lancet Glob Health.

[19] Ministry of Health and Population (2022).

[20] Ministry of Health and Population (2019).

[21] Ministry of Health and Population (2022).

[22] UNAIDS (2023).

[23] Ministry of Finance (2022).

[24] Institute for Health Metrics and Evaluation (IHME) (2024). Global Burden of Disease 2021: Findings from the GBD 2021 Study.

[25] World Bank (2024). Official exchange rate (LCU per US$, period average) - Nepal. https://data.worldbank.org/indicator/PA.NUS.FCRF?locations=NP.

[26] Husereau D., Drummond M., Augustovski F. (2022). Consolidated health economic evaluation reporting standards 2022 (CHEERS 2022) statement: updated reporting guidance for health economic evaluations. MDM Pol Pract.

[27] World Bank (2023). GDP per capita (current US$) - Nepal. https://data.worldbank.org/indicator/NY.GDP.PCAP.CD?locations=NP.

[28] Woods B., Revill P., Sculpher M., Claxton K. (2016). Country-level cost-effectiveness thresholds: initial estimates and the need for further research. Value Health.

[29] WHO (2012). Cost-effectiveness thresholds. http://www.who.int/choice/costs/CER_thresholds/en/index.html.

[30] Briggs A., Sculpher M., Claxton K. (2006).

[31] Nepal Planning Commission, Government of Nepal (2019).

[32] WHO (2024). Elimination of mother-to-child transmission HIV, hepatitis B and syphilis in Asia and the Pacific. https://www.aidsdatahub.org/thematic-areas/emtct/triple-emtct-data.

[33] Ministry of Health and Population, New ERA, ICF (2022). https://www.dhsprogram.com/pubs/pdf/SPA35/SPA35.pdf.

[34] Global Fund (2024).

[35] Sidibé M., Singh P.K. (2016). Thailand eliminates mother-to-child transmission of HIV and syphilis. Lancet.

[36] WHO (2019).

[37] UNAIDS (2024).

[38] Kitson A., Marshall A., Bassett K., Zeitz K. (2013). What are the core elements of patient-centred care? A narrative review and synthesis of the literature from health policy, medicine and nursing. J Adv Nurs.

[39] Bocoum F.Y., Tarnagda G., Bationo F. (2017). Introducing onsite antenatal syphilis screening in Burkina Faso: implementation and evaluation of a feasibility intervention tailored to a local context. BMC Health Serv Res.

