# Supplementary material for: Cost-effectiveness of integrated maternal HIV, syphilis, and hepatitis B screening opt-out strategies in Nepal: a modelling study
Source: Lancet Reg Health Southeast Asia. 2025 Jan 10;32:100524. doi: 10.1016/j.lansea.2024.100524 (PMC11758079; doi:10.1016/j.lansea.2024.100524)
Supplement: Supplementary Material [file mmc1.docx]

**Supplementary materials**

Cost-effectiveness of integrated maternal HIV, syphilis, and hepatitis B screening opt-out strategies in Nepal: a modelling study

Table of Contents

[Appendix 1: Abstract in Nepali 2](#_Toc183698197)

[Appendix 2: Schematic Markov model 3](#_Toc183698198)

[Figure 1 - Schematic of the Markov cohort model for HIV 3](#_Toc183698199)

[Figure 2 - Schematic of the Markov cohort model for syphilis 3](#_Toc183698200)

[Figure 3 - Schematic of the Markov cohort model for hepatitis B 3](#_Toc183698201)

[Appendix 3: Model parameters 4](#_Toc183698202)

[Table 1 – Model parameters 4](#_Toc183698203)

[Table 2 - Unit time and cost for antenatal screening for health workers 7](#_Toc183698204)

[Appendix 4: One-way sensitivity analysis 8](#_Toc183698205)

[Table 3 – One-way sensitivity analysis of ICER for dual-integrated screening for HIV compared with HIV screening only (status quo) and syphilis and triple-integrated screening for HIV, syphilis and hepatitis B compared with dual-integrated screening. 8](#_Toc183698206)

[Appendix 5: CHEERS 2022 Checklist 9](#_Toc183698207)

[References 11](#_Toc183698208)

# **Appendix 1: Abstract in Nepali**

**साराशं**

**पृष्ठभूमि**

एसियामा एचआईभी (HIV) को ठाडो संक्रमण (vertical transmission) को तेहोरो उन्मूलन (triple elimination) का लागि विश्व स्वास्थ्य संगठनले एकीकृत पद्दतिलाई प्रोत्साहित गर्ने एक बृहत खाकाको विकास गरेको छ। नेपालमा हालको स्क्रिनिङ् अभ्यास हेर्दा एचआईभीको तुलनामा सिफिलिस (Syphilis) र हेपाटाइटिस बी (Hepatitis B) को स्क्रीनिङ् को दायरा निकै कम देखिन्छ जसले एकीकृत रणनीतिको संभ्याव्यतालाई ईंगित गर्दछ। हामीले यस अध्ययनमा नेपालमा पूर्वप्रसूति सेवाको क्रममा तेहोरो स्क्रीनिङ् (triple screening) को लागत-प्रभावकारिताको मोडलिङ गर्ने लक्ष्य राखेका थियौं।

**विधि**

हामीले मातृत्व लक्षित एचआईभी, हेपाटाइटिस बी र सिफिलिस सेवाको नियमितता (cascade of care) र तिनीहरूसँग सम्बन्धित रोगको अवस्थालाई एक वर्षको चक्रिय प्रक्रिया र 20-वर्षको समय सीमाका आधारमा मार्कोभ मोडेल प्रयोग गरी रोग-विशेष मोडलिङ गर्यौ। साथै, एचआईभी र सिफिलिसको दोहोरो एकीकृत स्क्रीनिङ् तथा एचआईभी, सिफिलिस र हेपाटाइटिस बीको तेहोरो एकीकृत स्क्रीनिङ्लाई एचआईभी स्क्रिनिङ मात्रसँग तुलना गर्यौं। सो क्रममा लागतलाई सेवा प्रदायकको दृष्टिकोणबाट अनुमान गरिएको थियो। पन्छाइने प्रति असक्षमता-समायोजित जीवन वर्ष (DALYs) को सन्दर्भमा सिमान्तकृत लागत-प्रभावकारिता अनुपात (ICERs) लाई नतिजाको रुपमा प्रस्तुत गरियो। साथै एकल एवं सम्भाव्य संवेदनशीलता (univariable and probabilistic sensitivity) विश्लेषणहरू गरियो।

**निष्कर्ष**

हाम्रो मोडलिङ विश्लेषणले हाल अभ्यासमा रहेको एचआईभीका लागि मात्र गरिने पूर्वप्रसुति स्क्रिनिङको तुलनामा एचआईभी र सिफिलिसका लागि दोहोरो-एकीकृत स्क्रीनिङ् तथा एचआईभी, सिफिलिस र हेपाटाइटिस बीका लागि तेहोरो-एकीकृत पूर्वप्रसुति स्क्रिनिङको रणनीति अत्यधिक लागत-प्रभावकारी (अमेरिकी डलरमा ICERs 74 देखि 114 सम्म) हुने देखायो। यसका अतिरिक्त एकल-रणनीतिको तुलनामा दोहोरो- र तेहोरो-एकीकृत स्क्रीनिङ् रणनीतिका लागि क्रमशः 98% र 96% सम्भाव्य संवेदनशीलता विश्लेषण अनुमानहरू लागत-प्रभावाकारी हुने साबित भयो।

**व्याख्या**

प्राप्त नतिजाहरूले मातृ तथा नवजात शिशु रुग्णता कम गर्ने लक्ष्य राख्दै प्रारम्भिक पहिचान र सो अनुरुपको कार्यान्वयन (early detection and intervention) को माध्यमबाट नेपाल एवं बृहत्तर रुपमा एशियामा एकीकृत तेहोरो पूर्वप्रसुति स्क्रिनिङ् लागू गर्नका लागि विश्व स्वास्थ्य संगठनका सिफारिसहरूलाई समर्थन गर्दछ।

# **Appendix 2: Schematic Markov model**

## **Figure 1 - Schematic of the Markov cohort model for HIV**


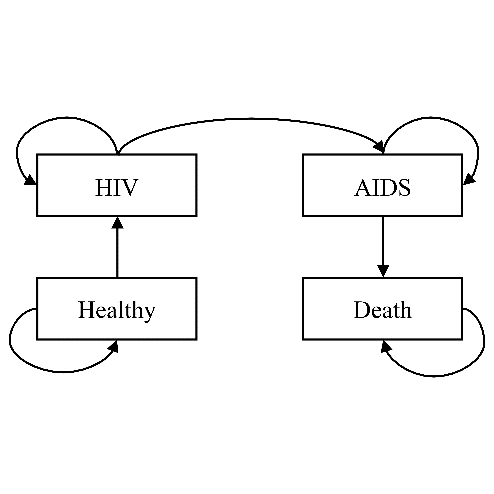


Figure 1 presents a schematic of the Markov progression states for HIV, adapted from Owusu-Edusei et al ^1^. State transition probabilities were obtained from the literature (see Appendix 2, Table 1). We assumed that patients can only remain in the same state or progress; it is not feasible for them to move back to a less severe state.

## **Figure 2 - Schematic of the Markov cohort model for syphilis**


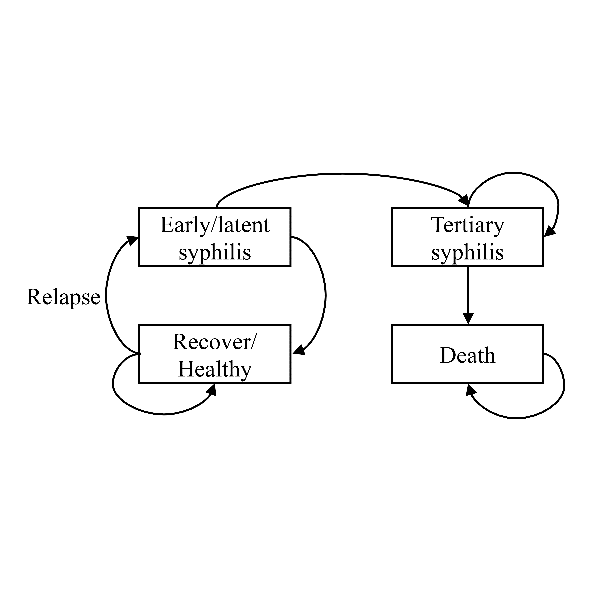


Figure 2 presents a schematic of the Markov progression states for syphilis, adopted from Owusu-Edusei et al ^1^. State transition probabilities were obtained from the literature (see Appendix 2, Table 1). For treated women, there is a possibility of syphilis relapse at t=1. We assumed no relapse possible for children. For untreated infants with congenital syphilis, the disability weight was applied to the first three years of life, after which the children will move into the early/secondary syphilis stage and then follow the transitions from one state to the other in the mother's model.

## **Figure 3 - Schematic of the Markov cohort model for hepatitis B**


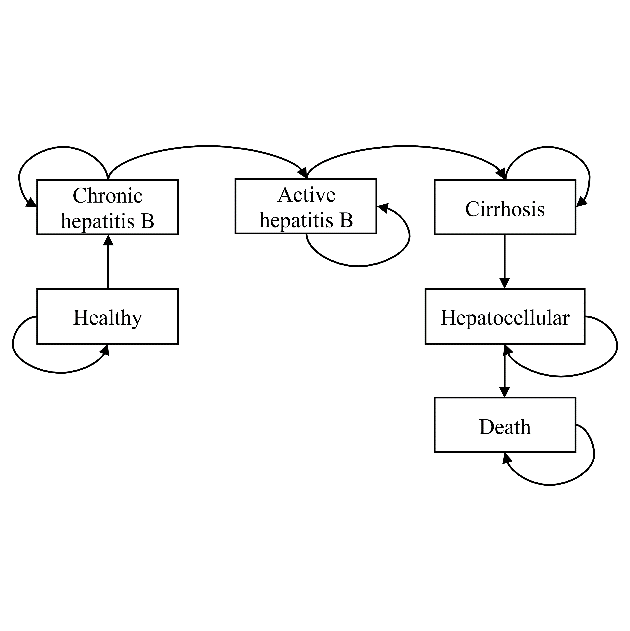


Figure 3 illustrates a schematic of the Markov progression states for hepatitis B, adopted from Su et al ^2^. State transition probabilities were obtained from the literature (see Appendix 2, Table 1). We assumed that patients can only remain in the same state or progress; it is not feasible for them to move back to a less severe state. We assumed that treated women stayed in state B all their lives.

# **Appendix 3: Model parameters**

## **Table 1 – Model parameters**

| **Description** | **Base** | **Lower** | **Upper** | **References** |
| --- | --- | --- | --- | --- |
| *Transition probabilities for HIV* |  |  |  |  |
| Transition probability from HIV to AIDS for mothers if treatment | 0.006 | 0.003 | 0.010 | ^1^ |
| Transition probability from HIV to AIDS for mothers if no treatment | 0.060 | 0.030 | 0.090 | ^1^ |
| Transition probability from AIDS to death for mothers if no treatment | 0.030 | 0.015 | 0.045 | ^1^ |
| *Transition probabilities for hepatitis B* |  |  |  |  |
| Transition probability from chronic hepatitis B to active hepatitis B if no treatment | 0.002 | 0.001 | 0.003 | ^2^ |
| Transition probability from active hepatitis B to cirrhosis if no treatment | 0.029 | 0.015 | 0.058 | ^2^ |
| Transition probability from active hepatitis B to hepatocellular if no treatment | 0.002 | 0.001 | 0.003 | ^2^ |
| Transition probability from hepatocellular to death if no treatment | 0.450 | 0.220 | 0.700 | ^2^ |
| Transition probability from cirrhosis to hepatocellular if no treatment | 0.034 | 0.010 | 0.100 | ^2^ |
| Transition probability from cirrhosis to death if no treatment | 0.031 | 0.030 | 0.038 | ^2^ |
| *Transition probabilities for syphilis* |  |  |  |  |
| Transition probability from early or latent to tertiary syphilis if no treatment | 0.330 | 0.165 | 0.495 | ^1^ |
| Transition probability from tertiary syphilis to death if no treatment | 0.110 | 0.055 | 0.165 | ^1^ |
| Transition probability from cured to early or latent syphilis after treatment (relapse) | 0.236 | 0.118 | 0.354 | ^1^ |
| *HIV-related costs* |  |  |  |  |
| Screening for mother ($/rapid test) |  |  |  |  |
| Treatment for mother ($/year) | 45.00 | 40.50 | 49.50 | ^3^ |
| Screening for newborn ($/rapid test) | 1.12 | 1.00 | 1.23 | ^3^ |
| Treatment for children up to 15 years old ($/year) | 238.79 | 214.91 | 262.67 | ^3^ |
| *Hepatitis B-related costs* |  |  |  |  |
| Screening ($/rapid test) | 0.78 | 0.70 | 0.86 | ^3^ |
| Confirmatory test ($/ELISA test) | 1.47 | 1.32 | 1.62 | ^4^ |
| Treatment for mother ($/year) | 28.80 | 25.92 | 31.68 | ^3^ |
| Treatment for newborn ($/injection) | 35.43 | 31.89 | 38.97 | ^5^ |
| *Syphilis-related costs* |  |  |  |  |
| Integrated syphilis/HIV screening ($/rapid test) | 1.23 | 1.11 | 1.35 | ^3^ |
| Confirmatory test ($/TPPA) | 3.60 | 3.24 | 3.96 | ^5^ |
| Treatment for mother ($/injection) | 0.15 | 0.14 | 0.17 | ^4^ |
| Congenital syphilis treatment ($/injection) | 0.15 | 0.14 | 0.17 | ^4^ |
| Congenital syphilis additional costs for birth complications ($/newborn) | 15.00 | 13.50 | 16.50 | ^6^ |
| Neonatal death and stillbirths ($/event) | 33.75 | 30.38 | 37.13 | ^6^ |
| *HIV-related DALY weights* |  |  |  |  |
| HIV | 0.143 | 0.006 | 0.377 | ^7^ |
| AIDS, no treatment | 0.582 | 0.406 | 0.743 | ^7^ |
| AIDS, with treatment | 0.078 | 0.052 | 0.111 | ^7^ |
| *Hepatitis B-related DALY weights* |  |  |  |  |
| Chronic hepatitis B | 0.051 | 0.032 | 0.074 | ^7^ |
| Active hepatitis B | 0.133 | 0.088 | 0.190 | ^7^ |
| Cirrhosis | 0.220 | 0.123 | 0.404 | ^7^ |
| Hepatocellular carcinoma | 0.332 | 0.031 | 0.687 | ^7^ |
| *Syphilis-related DALY weights* |  |  |  |  |
| Early/Latent syphilis | 0.006 | 0.002 | 0.012 | ^7^ |
| Tertiary syphilis | 0.310 | 0.032 | 0.669 | ^7^ |
| Congenital syphilis (3 years) | 0.048 | 0.001 | 0.290 | ^7^ |
| *Other HIV-related parameters* |  |  |  |  |
| Prevalence of HIV in pregnant women | 0.0012 | 0.0010 | 0.0014 | ^8^ |
| Probability of being AIDS infected when HIV-positive at t=0 | 0.206 | 0.165 | 0.247 | ^8^ |
| Vertical transmission probability in the absence of treatment | 0.215 | 0.172 | 0.258 | ^9^ |
| Vertical transmission probability if treatment | 0.02 | 0.016 | 0.024 | ^10^ |
| Duration of HIV with treatment. newborn | 15 | 7.5 | 22.5 | ^1^ |
| Duration of HIV without treatment, newborn | 2 | 1 | 3 | ^1^ |
| Duration of AIDS with treatment, child | 5 | 2.5 | 7.5 | ^1^ |
| Duration of AIDS without treatment, child | 1 | 0.5 | 1.5 | ^1^ |
| Probability of a pregnant woman being screened | 0.82 | 0.656 | 0.984 | ^11^ Screening rate used in the model was based on actual HIV screening data from the National Centre for AIDS And STD Control that already takes into account opt-out screening and treatment. As a result, the opt-out rate was implicitly included in our analysis. |
| Probability of a child born to an HIV-positive woman being screened | 0.927 | 0.742 | 1 | ^8^ |
| Sensitivity of HIV rapid test | 0.999 | 0.9 | 1 | ^12^ |
| Specificity of HIV rapid test | 0.999 | 0.9 | 1 | ^12^ |
| Probability for an HIV-positive pregnant woman to receive treatment | 0.77 | 0.67 | 0.89 | ^13^ |
| Probability for an HIV-positive newborn to receive treatment | 0.77 | 0.67 | 0.89 | ^13^ |
| HIV treatment effect | 0.1 | 0.08 | 0.12 | ^1^ |
| *Other hepatitis B-related parameters* |  |  |  |  |
| Prevalence of hepatitis B in pregnant women | 0.005 | 0.004 | 0.006 | ^14^ |
| Vertical transmission probability from mothers with chronic hepatitis B, no PMTCT | 0.25 | 0.1 | 0.4 | ^15^ |
| Vertical transmission probability from mothers with active hepatitis B, no PMTCT | 0.8 | 0.7 | 0.9 | ^15^ |
| Vertical transmission probability, PMTCT | 0.01 | 0.00 | 0.05 | ^15^ |
| Treatment coverage for mothers and children | 0.77 | 0.67 | 0.89 | As there is no official data on treatment coverage for hepatitis B in Nepal, we used HIV treatment coverage as a proxy, reflecting the integrated approach proposed in our model. |
| Sensitivity of hepatitis B rapid test | 0.900 | 0.891 | 0.908 | ^2^ |
| Specificity of hepatitis B rapid test | 0.995 | 0.994 | 0.995 | ^2^ |
| Sensitivity of hepatitis B confirmatory test (ELISA) | 0.932 | 0.851 | 0.985 | ^2^ |
| Specificity of hepatitis B confirmatory test (ELISA) | 0.931 | 0.851 | 0.999 | ^2^ |
| *Other syphilis-related parameters* |  |  |  |  |
| Prevalence of syphilis in pregnant women | 0.0016 | 0.0013 | 0.0019 | ^16^ |
| Probability that syphilis infection is at the latent stage at t=0 | 0.75 | 0.6 | 0.9 | ^1^ |
| Vertical transmission probability of foetal transmission if early syphilis | 0.5 | 0.4 | 0.6 | ^1^ |
| Vertical transmission probability of foetal transmission if latent syphilis | 0.70 | 0.56 | 0.84 | ^1^ |
| Probability of stillbirth given foetal transmission | 0.21 | 0.168 | 0.252 | ^1^ |
| Probability of neonatal death given foetal transmission | 0.09 | 0.07 | 0.11 | ^1^ |
| Probability of a pregnant woman receiving screening | 0.003 | 0.0024 | 0.0036 | ^17^ |
| Sensitivity of syphilis rapid test | 0.9 | 0.8 | 1 | ^1^ |
| Specificity of syphilis rapid test | 0.957 | 0.9 | 1 | ^1^ |
| Sensitivity of syphilis confirmatory test (TPPA) | 0.92 | 0.8 | 1 | ^1^ |
| Specificity of syphilis confirmatory test (TPPA) | 0.99 | 0.98 | 1 | ^1^ |
| Treatment coverage for mothers and children | 0.77 | 0.67 | 0.89 | As there is no official data on treatment coverage for syphilis in Nepal, we used HIV treatment coverage as a proxy, reflecting the integrated approach proposed in our model. |
| Probability of treatment success | 0.95 | 0.76 | 1 | ^1^ |
| *Other parameters* |  |  |  |  |
| Probability for a pregnant woman to receive integrated screening for HIV and syphilis | 0.82 | 0.656 | 0.984 | Assumed that integration will achieve the same coverage as HIV screening alone. |
| Probability for a pregnant woman to receive integrated screening for HIV, syphilis and hepatitis B | 0.82 | 0.656 | 0.984 | Assumed that integration will achieve the same coverage as HIV screening alone. |
| Annual discount rate - costs | 0.03 | 0.00 | 0.06 | ^1^ |
| Annual discount rate - benefits | 0.03 | 0.00 | 0.06 | ^1^ |
| Mothers’ life expectancy at birth (year) | 65 | 52.0 | 78.0 | ^18^ |
| Babies’ life expectancy at birth (year) | 68 | 54.4 | 81.6 | ^18^ |
| Number of pregnant women (per year) | 752506 | 602005 | 903007 | ^19^ |
| Number of pregnant women with HIV under treatment at the time of their pregnancy | 165 | NA | NA | ^13^ |
| Number of required ANC visits | 4 | NA | NA | ^20^ |
| Weeks of the first ANC visits | 12 | 9.6 | 14.4 | ^20^ |
| ANC coverage of at least 1 visit | 0.84 | 0.67 | 1.00 | ^20^ |
| Median age at first birth for women aged 25-49 (year) | 21 | 16.8 | 25.2 | ^21^ |
| Hourly rate of nurses | 1.20 |  |  | ^22^ |
| Hourly rate of doctors | 2.12 |  |  | ^22^ |
| Conversion rate of Nepalese rupees in dollars | 0.0075 | 0.0060 | 0.0090 | ^23^ |

## **Table 2 - Unit time and cost for antenatal screening for health workers**

| **Variables** | **HIV only** | **HIV and syphilis** | | **HIV, syphilis and hepatitis B** | |
| --- | --- | --- | --- | --- | --- |
| **Time (hours)** |  |  | |  | |
| ***Administration*** |  |  | |  | |
| Data entry at health facility (per record) | 0.25 | 0.50 | | 0.67 | |
| ***Training and supervision (every five years)*** |  |  | |  | |
| Training | 21.00 | 45.00 | | 60.00 | |
| Supervision of training | 36.00 | 75.00 | | 105.00 | |
| ***Pregnancy screening and treatment*** |  |  | |  | |
| Counselling and testing | 0.50 | 0.67 | | 1.00 | |
| Confirmation of diagnosis | 0.50 | 0.50 | | 0.50 | |
| Treatment for adults | 0.50 | 0.50 | | 0.50 | |
| ***Postpartum treatment*** |  |  | |  | |
| Treatment for exposed newborns | 0.50 | 0.50 | | 0.50 | |
|  |  |  | |  | |
| **Workforce cost ($)** |  |  | |  | |
| ***Administration (per record)*** |  |  | |  | |
| Data entry at health facility | 0.30 | 0.60 | | 0.80 | |
| ***Training supervision (per year)*** |  |  | |  | |
| Training | 5.02 | 10.77 | | 14.36 | |
| Supervision of training | 15.27 | 31.81 | | 44.54 | |
| Training costs | 20.29 | 42.58 | | 58.89 | |
| ***Pregnancy screening and treatment (per woman)*** | | |  | |  |
| Counselling and testing | 0.60 | 0.80 | | 1.20 | |
| Confirmation | 0.60 | 0.60 | | 0.60 | |
| Treatment for adults | 0.60 | 0.60 | | 0.60 | |
| ***Postpartum treatment (per newborn)*** |  |  | |  | |
| Treatment for exposed newborns | 0.60 | 0.60 | | 0.60 | |

We assumed that nurses were responsible for most tasks, including administration, receiving training, screening, confirmation and delivering treatment. Doctors were responsible for training supervision. We assume that training takes place once every five years. Nurses' and doctors’ incomes were estimated based on the new salary scale for the public sector. See Supplementary Material (Appendix 2, Table 1) for parameter details. The time required to prescribe treatment, carry out screening and attend training sessions was based on a cost-efficiency analysis of the triple-integrated screening carried out in Cambodia ^15^. For the children tested, we did not take into account the cost of training, as this was already included in the mother's test, and newborns could only be tested if the mother was.

# **Appendix 4: One-way sensitivity analysis**

## **Table 3 – One-way sensitivity analysis of ICER for dual-integrated screening for HIV compared with HIV screening only (status quo) and syphilis and triple-integrated screening for HIV, syphilis and hepatitis B compared with dual-integrated screening.**

|  | **HIV and syphilis** | | **HIV, syphilis and hepatitis B** | |
| --- | --- | --- | --- | --- |
|  | Lower | Upper | Lower | Upper |
| HIV drug cost (mother) | 18 | 18 | 114 | 114 |
| HIV drug cost (child) | 18 | 18 | 114 | 114 |
| Integrated syphilis/HIV screening kit cost | 16 | 21 | 114 | 114 |
| Syphilis drug cost (mother) | 18 | 18 | 114 | 114 |
| Syphilis drug cost (child) | 18 | 18 | 114 | 114 |
| Hepatitis B screening kit cost | 18 | 18 | 108 | 120 |
| Hepatitis B drug cost (mother) | 18 | 18 | 108 | 120 |
| Hepatitis B drug cost (child) | 18 | 18 | 114 | 114 |
| Costs annual discount rate | 18 | 18 | 199 | 86 |
| Benefits annual discount rate | 16 | 20 | 1148 | 75 |
| Prevalence of HIV | 18 | 18 | 114 | 114 |
| Prevalence of hepatitis B | 18 | 18 | 138 | 97 |
| Prevalence of syphilis | 23 | 15 | 114 | 114 |
| Hourly rate of nurses | 17 | 20 | 114 | 113 |
| Hourly rate of doctors | 18 | 18 | 114 | 114 |

Notes: HIV= Human immunodeficiency virus. NA= Not applicable. ICER= Incremental cost-effectiveness ratio.

# **Appendix 5: CHEERS 2022 Checklist**

| **Topic** | **No.** | **Item** | **Location where item is reported** |
| --- | --- | --- | --- |
| **Title** |  |  |  |
|  | 1 | Identify the study as an economic evaluation and specify the interventions being compared. | p1 |
| **Abstract** |  |  |  |
|  | 2 | Provide a structured summary that highlights context, key methods, results, and alternative analyses. | p2 |
| **Introduction** |  |  |  |
| **Background and objectives** | 3 | Give the context for the study, the study question, and its practical relevance for decision making in policy or practice. | p4-5 |
| **Methods** |  |  |  |
| **Health economic analysis plan** | 4 | Indicate whether a health economic analysis plan was developed and where available. | p5 |
| **Study population** | 5 | Describe characteristics of the study population (such as age range, demographics, socioeconomic, or clinical characteristics). | p5 |
| **Setting and location** | 6 | Provide relevant contextual information that may influence findings. | p5-8 |
| **Comparators** | 7 | Describe the interventions or strategies being compared and why chosen. | p7 |
| **Perspective** | 8 | State the perspective(s) adopted by the study and why chosen. | p8 |
| **Time horizon** | 9 | State the time horizon for the study and why appropriate. | p5 |
| **Discount rate** | 10 | Report the discount rate(s) and reason chosen. | p7 |
| **Selection of outcomes** | 11 | Describe what outcomes were used as the measure(s) of benefit(s) and harm(s). | p8 |
| **Measurement of outcomes** | 12 | Describe how outcomes used to capture benefit(s) and harm(s) were measured. | p8 |
| **Valuation of outcomes** | 13 | Describe the population and methods used to measure and value outcomes. | p5-7 |
| **Measurement and valuation of resources and costs** | 14 | Describe how costs were valued. | p8 |
| **Currency, price date, and conversion** | 15 | Report the dates of the estimated resource quantities and unit costs, plus the currency and year of conversion. | p8 and supplementary materials |
| **Rationale and description of model** | 16 | If modelling is used, describe in detail and why used. Report if the model is publicly available and where it can be accessed. | p5-7 |
| **Analytics and assumptions** | 17 | Describe any methods for analysing or statistically transforming data, any extrapolation methods, and approaches for validating any model used. | p8-9 |
| **Characterising heterogeneity** | 18 | Describe any methods used for estimating how the results of the study vary for subgroups. | p8-9 |
| **Characterising distributional effects** | 19 | Describe how impacts are distributed across different individuals or adjustments made to reflect priority populations. | P5-7 |
| **Characterising uncertainty** | 20 | Describe methods to characterise any sources of uncertainty in the analysis. | p8-9 |
| **Approach to engagement with patients and others affected by the study** | 21 | Describe any approaches to engage patients or service recipients, the general public, communities, or stakeholders (such as clinicians or payers) in the design of the study. | NA |
| **Results** |  |  |  |
| **Study parameters** | 22 | Report all analytic inputs (such as values, ranges, references) including uncertainty or distributional assumptions. | Supplementary Materials, Table 1 |
| **Summary of main results** | 23 | Report the mean values for the main categories of costs and outcomes of interest and summarise them in the most appropriate overall measure. | p9 |
| **Effect of uncertainty** | 24 | Describe how uncertainty about analytic judgments, inputs, or projections affect findings. Report the effect of choice of discount rate and time horizon, if applicable. | p9-13 |
| **Effect of engagement with patients and others affected by the study** | 25 | Report on any difference patient/service recipient, general public, community, or stakeholder involvement made to the approach or findings of the study | NA |
| **Discussion** |  |  |  |
| **Study findings, limitations, generalisability, and current knowledge** | 26 | Report key findings, limitations, ethical or equity considerations not captured, and how these could affect patients, policy, or practice. | p14-16 |
| **Other relevant information** |  |  |  |
| **Source of funding** | 27 | Describe how the study was funded and any role of the funder in the identification, design, conduct, and reporting of the analysis | p17 |
| **Conflicts of interest** | 28 | Report authors conflicts of interest according to journal or International Committee of Medical Journal Editors requirements. | p17 |

*From:* Husereau D, Drummond M, Augustovski F, et al. Consolidated Health Economic Evaluation Reporting Standards 2022 (CHEERS 2022) Explanation and Elaboration: A Report of the ISPOR CHEERS II Good Practices Task Force. Value Health 2022;25. <doi:10.1016/j.jval.2021.10.008>

# **References**

1 Owusu-Edusei Jr K, Tao G, Gift TL, *et al.* Cost-effectiveness of integrated routine offering of prenatal HIV and syphilis screening in China. *Sexually Transmitted Diseases* 2014; **41**: 103–10.

2 Su S, Wong WC, Zou Z, *et al.* Cost-effectiveness of universal screening for chronic hepatitis B virus infection in China: an economic evaluation. *The Lancet Global Health* 2022; **10**: e278–87.

3 Global Fund. Pooled Procurement Mechanism Reference Pricing. 2024.

4 MSH, WHO. International Medical Products Price Guide. 2023.

5 Global Fund. Price and quality reference reports. 2024.

6 NHSSP. Aama Programme: a programme for Nepali women. 2014.

7 IHME. Global Burden of Disease Study 2021. 2021.

8 UNAIDS. Country factsheets, Nepal Data. 2020.

9 Rollins N, Mahy M, Becquet R, Kuhn L, Creek T, Mofenson L. Estimates of peripartum and postnatal mother-to-child transmission probabilities of HIV for use in Spectrum and other population-based models. *Sexually transmitted infections* 2012; **88**: i44–51.

10 Chibwesha CJ, Giganti MJ, Putta N, *et al.* Optimal time on HAART for prevention of mother-to-child transmission of HIV. *JAIDS Journal of Acquired Immune Deficiency Syndromes* 2011; **58**: 224–8.

11 NCASC. HIV Factsheet. 2022.

12 Rodriguez PJ, Roberts DA, Meisner J, *et al.* Cost-effectiveness of dual maternal HIV and syphilis testing strategies in high and low HIV prevalence countries: a modelling study. *The Lancet Global Health* 2021; **9**: e61–71.

13 UNAIDS. Country factsheets, Nepal. 2023.

14 Ministry of Health and Population. National Strategy for Viral Hepatitis B and C. 2023.

15 Zhang L, Tao Y, Woodring J, *et al.* Integrated approach for triple elimination of mother-to-child transmission of HIV, hepatitis B and syphilis is highly effective and cost-effective: an economic evaluation. *International journal of epidemiology* 2019; **48**: 1327–39.

16 Shakya S, Thingulstad S, Syversen U, *et al.* Prevalence of sexually transmitted infections among married women in rural Nepal. *Infectious diseases in obstetrics and gynecology* 2018; **2018**.

17 Trivedi S, Taylor M, Kamb ML, Chou D. Evaluating coverage of maternal syphilis screening and treatment within antenatal care to guide service improvements for prevention of congenital syphilis in Countdown 2030 Countries. *Journal of Global Health* 2020; **10**.

18 World Bank. Life expectancy at birth. 2021.

19 Nepal Planning Commission, Government of Nepal. Multiple Indicator Cluster Survey (MICS). 2019.

20 Ministry of Health and Population. Nepal Safe Motherhood and Newborn Health Road Map 2030. 2019.

21 Ministry of Health and Population. Nepal Demographic and Health Survey 2022. 2022.

22 Ministry of Finance. Salary and grade grid. 2022.

23 World Bank. Official exchange rate (LCU per US$, period average) - Nepal. 2024. https://data.worldbank.org/indicator/PA.NUS.FCRF?locations=NP.
